# Supplementary material for: Long-Term Longitudinal Analysis of Neutralizing Antibody Response to Three Vaccine Doses in a Real-Life Setting of Previously SARS-CoV-2 Infected Healthcare Workers: A Model for Predicting Response to Further Vaccine Doses
Source: Vaccines (Basel). 2022 Aug 2;10(8):1237. doi: 10.3390/vaccines10081237 (PMC9416151; doi:10.3390/vaccines10081237)
Supplement: Supplementary file 1 [file vaccines-10-01237-s001.zip › vaccines-1822496-supplementary.pdf]

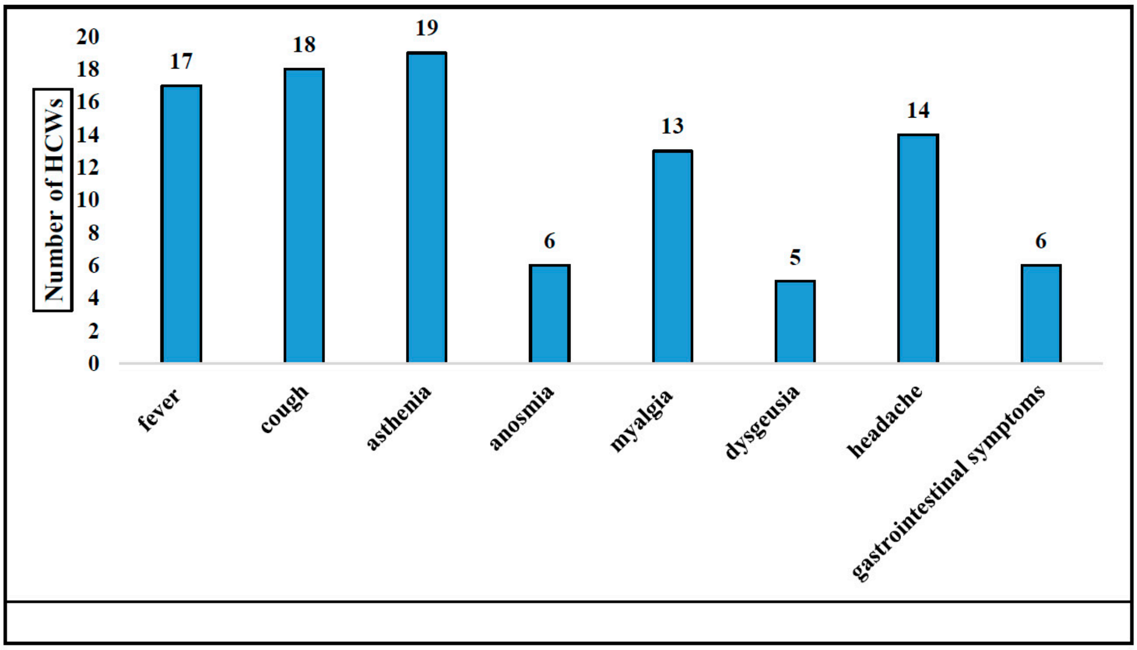

Figure S1. Signs and/or symptoms of the 19 healthcare workers (HCWs) with mild COVID-19 disease. Data are expressed as absolute number of HCWs reporting the sign or symptom..

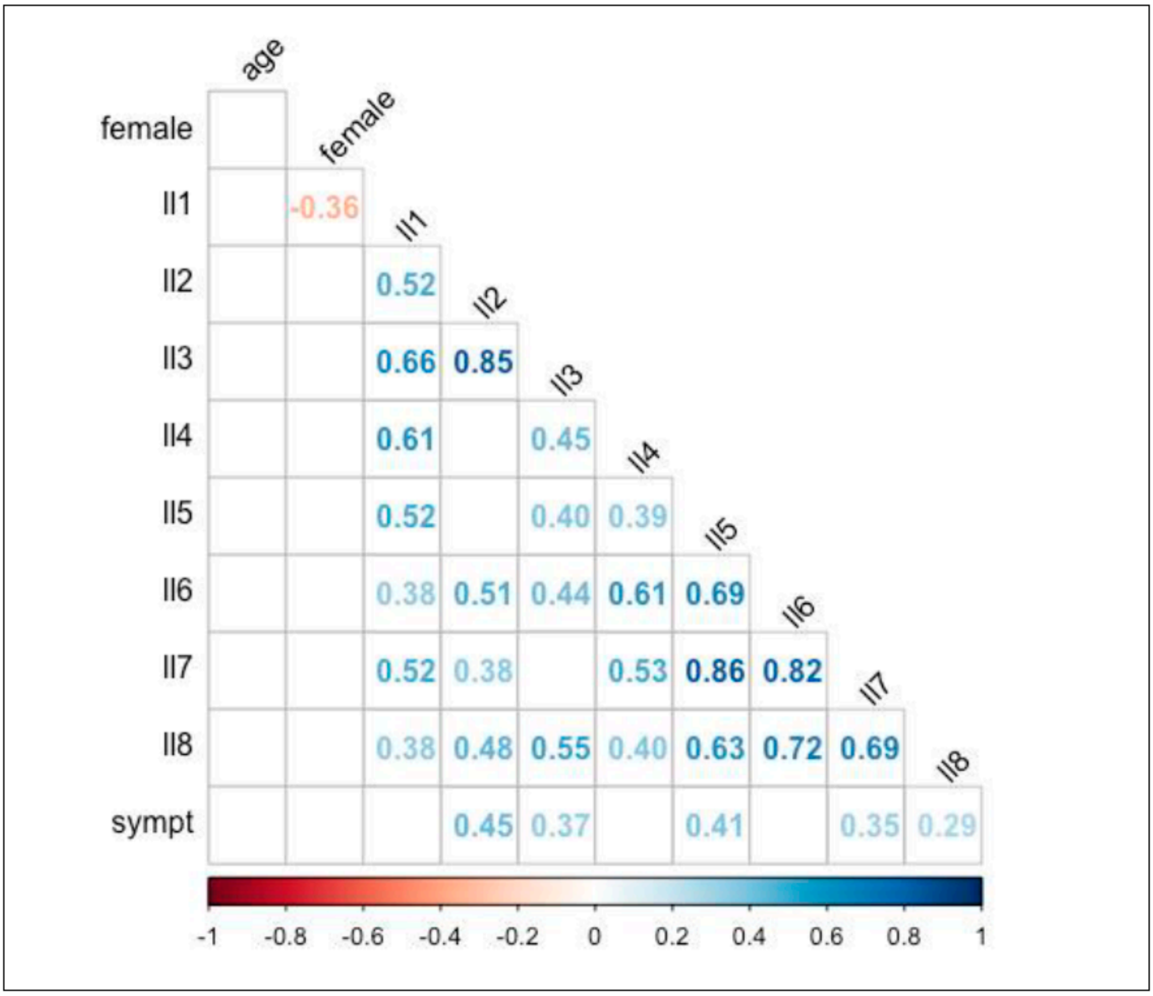

Figure S2. Pairwise correlations between NtAb titers (expressed as ID_{50}) at the eight study points, age, gender (expressed as being of female gender) and having a symptomatic disease. Rank method of Spearman was applied. Only significant Rho coefficients are reported. Blue: positive correlations, red: negative correlations. NtAb: neutralizing antibodies; II: Time; ID50: reciprocal value of the sample dilution that showed a 50% protection of virus cyto-pathic effect.
